# Development and validation of the Nurse Team Resilience Scale (NTRS) in the context of public health emergencies

**DOI:** 10.1186/s12912-023-01627-9

**Published:** 2023-12-20

**Authors:** Ya Su, Lin Wang, Tangyu Chen, Liwen Liao, SanLian Hu, Yan Yang

**Affiliations:** 1 School of Nursing, Shanghai Jiao Tong University, 227 S Chongqing Rd, Shanghai, 200025 China; 2 Department of Nursing, Shanghai Jiao Tong University Affiliated Sixth People’s Hospital, 600 Yishan Rd, Xuhui District, Shanghai, China; 3 Renji Hospital, Shanghai Jiao Tong University School of Medicine, No.1630 Dongfang Road, Pudong New District, Shanghai, China

**Keywords:** Team resilience, Nurses, Public health emergencies, Scale development, Instrument validation study

## Abstract

**Background:**

Team resilience can help nurse to respond positively to adversity at work and maintain normal team function in complex and unstable environments. However, much less research attention has been paid to team resilience than to individual resilience, and nurses lack reliable and valid tools to measure team resilience. This study aimed to develop and evaluate the psychometric properties of a scale that measures the nursing team resilience in the context of a public health emergency.

**Methods:**

The study was conducted in three stages that item development, scale development, and scale evaluation. This scale was based on that of Morgan and Sharma et al. proposed four-factor team resilience model, and the draft scale was generated based on the literature review, existing scales, experts’ validations, and cognitive interviews. During July 2022 to August 2022, the construct validity and the internal consistency reliability of the NTRS were evaluated through an online survey of 421 nurses.

**Results:**

The 8-item NTRS scale has good reliability and validity and is suitable for measuring the nurse team resilience. The EFA found a common factor solution and explained 72.33% of the common varianc and the CFA score showed construct validity. Reliability of the internal consistency of the scale with a good Cronbach alpha of 0.94.

**Conclusion:**

This scale can assess team resilience in nurses that nursing education and management resources can be allocated to improve policies and training programs to provide effective positive support to nurses in challenging workplace situations and to enable greater health systems resilience in the future.

**Supplementary Information:**

The online version contains supplementary material available at 10.1186/s12912-023-01627-9.

## Background

In times of global development and change, emergencies and adverse events may be inevitable, such as accidents, emergencies, high work demands, and so on. These events not only take a toll on employees mentally and physically, but can also negatively impact employee and team performance [1]. Various studies have shown that team resilience is an essential protective factor for both team and employee that can help both sides cope with challenging situations positively and maintain normal team function in this volatility, uncertainty, complexity, and ambiguity environment [2]. It is reported by the World Health Organization [3] that the nursing team is a vital part of the healthcare system that accounts for 59% of health professionals. It is essential to know about the level of team resilience for nurses which has an impact on patient safety and even the operation of the medical system [4, 5]. But less attention has been paid to team resilience than to individual resilience, and there is a lack of relevant measurement instruments for team resilience in nurse teams. Hence, it is crucial to develop a reliable scale to test the resilience of nurses team.

The concept of team resilience is diverse and lacks consensus. Team resilience is known to be defined as the ability to help a team recover from failure, setback, conflict, or other threats to team well-being, as suggested by West et al. [6]. Besides Morgan et al. [7] describe it as a dynamic psychosocial process that protects a group of individuals from the potential negative effects of the stressors they experience together and highlights the dynamic properties of team resilience. Many researchers have developed measurement instruments for team resilience in the context of corporates and elite sports based on the above definitions [8–13], and only one scale is developed for the background of crisis rescue [14]. Rare scales on team resilience are developed for nursing staff. On the other hand, the extensive literature shows that there is a lack of brief assessment tool to measure team resilience while most of the existing scales have more than 30 items [10, 11, 14]. It is crucial to have a brief and easy-use instrument that served as a screening tool for improving team resilience.

Public health emergencies are regarded as major threats to the health system, including terrorist attacks, infectious disease epidemics, and natural disasters. In public health emergencies, the nursing staff is an essential response team, a robust nursing workforce is needed necessarily for hospitals to cope with public health emergencies [15, 16]. A studies in China showed that the moderate level of nurses were 63.77 ± 12.80 [17]. Studies in Iran and Lebanon show that the moderate level of nurses during the COVID-19 period were 63.8 ± 16.2 and 72 ± 13.5 respectively [18, 19]. An Australian study showed that operating room nurses have higher mean resilience levels were 75.90 ± 11.00 [20]. A study in the United States showed that U.S. Air Force registered nurses have higher mean resilience levels were 77.00 ± 12.03 [21]. Most nurses measure resilience using the Connor Davidson Resilience Scale, however, it is able to distinguish resilient individuals from non-resilient individuals [22]. Consider that nurses have different levels of individual resilience in different scenarios and events. Therefore, a team resilience scale is required to help nursing teams respond positively to workplace adversity, thereby serving as a starting point for improving team resilience and helping organizations develop strategies that may improve team efficiency and stability. To our knowledge, there is currently no team resilience scale applicable to the nursing field.

The purpose of this study was to develop the Nurse Team Resilience Scale (NTRS) based on the four-factor team resilience model by using previous scales, literature review, expert validation, and cognitive interviews, and to evaluate the psychometric properties of the scale.

## Methods

### Phases and Procedure

**Fig. 1:**
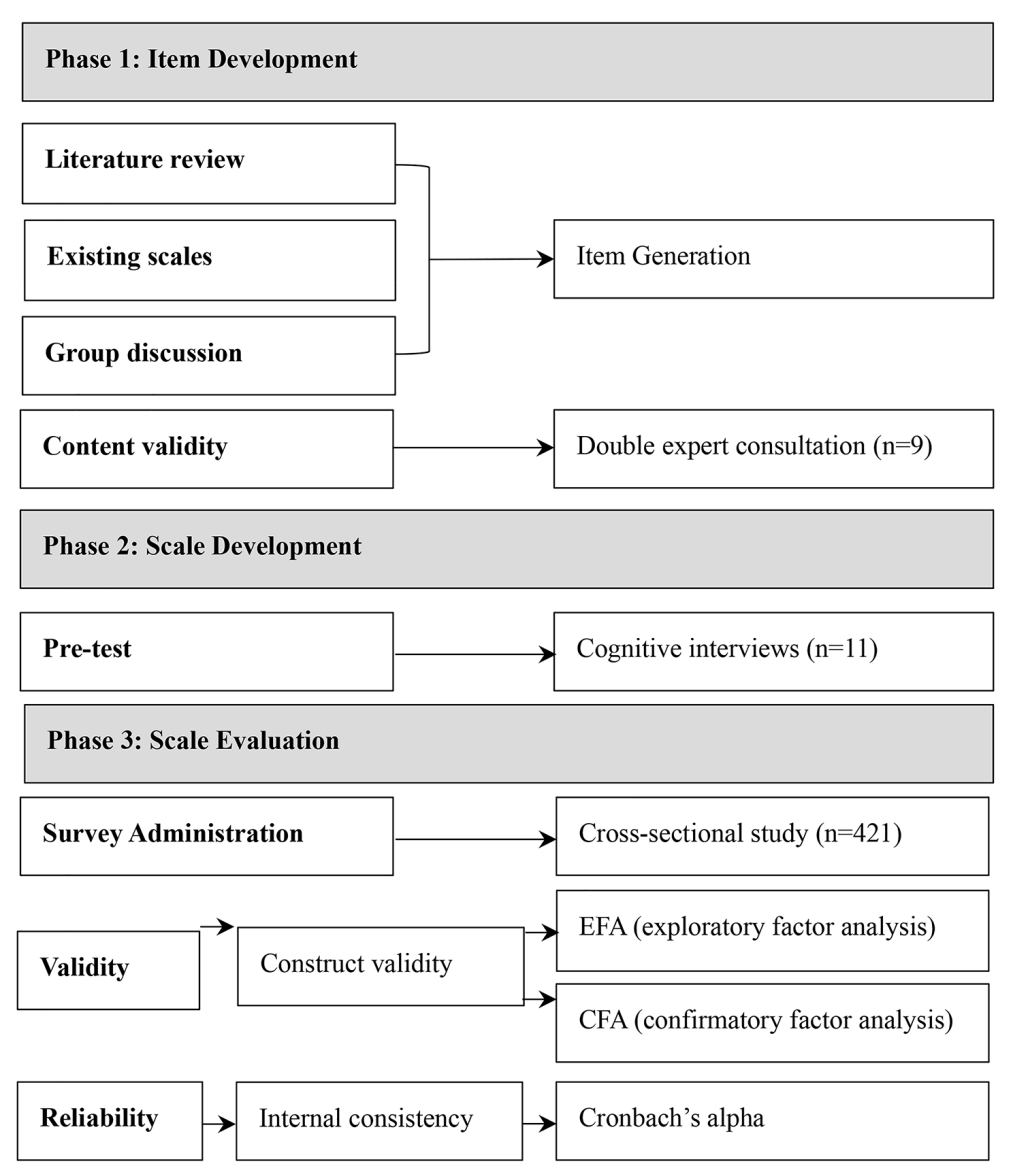
Overview of the three phases of scale development and validation

This methodological study was conducted in three phases [23–25]: (i) item development: the development of the NTRS through literature, existing scales, and group discussion; and content validation, by use of two-round expert consultation; (ii) development of the scales: pretest with cognitive interviews; and (iii) scale evaluation: psychometric testing of the scale by testing construct validity, criterion validity, and reliability (Fig. 1).

### Phase 1: Item Development

#### Identification of the Domain

After reviewing the literature, the framework proposed by Morgan et al. [23] is one of the most extensive theories for developing the scale of team resilience, which is comprised of four dimensions: team structure, approaches to dominance, social capital, and team effectiveness. Group structure means the rules for the formation of group norms and group roles, which consist of three structural characteristics: task design and composition, as well as group norms [10, 26]. Mastery approaches reflect how team members overcome difficulties and recover from adversity through their attitude and behavior [27, 28]. Social capital represents high-quality interaction and care relationships inside the team [29, 30]. Collective effectiveness reflects the team’s collective belief in its ability to get the job done [31].

#### Item generation

Searches were performed with keywords: ‘resilien*’ and ‘team’ in Pubmed, Web of Science, Scopus, CINAHL, PsycINFO, Business Source Complete, ProQuest Dissertations, CNKI, Wanfang Database, and VIP Database. Several scales [8, 10, 14] to measure team resilience were selected based on a literature review. Additionally, experts in mental health and nursing management were participated to help identify relevant issues and an initial set of items was selected to measure team resilience through focus group discussions. After developed, discussed, and refined possible designs, and then carefully selected 16 designs from the existing scale (Table 1). The questionnaire was evaluated using a 5-point Likert scale (from “strongly disagree” to “strongly agree”) [25].

**Table 1:** Initial development of the instrument

No.	Items generated	Factor	Source
1	Your team can set work goals with full energy.	Collective efficacy	CRTRQ
2	Your team can effectively achieve work goals.	Collective efficacy	CRTRQ
3	Your team can grow positively in the process of overcoming difficulties.	Collective efficacy	ETRS
4	Your team regards participation in work tasks as a high honor.	Collective efficacy	CRTRQ
5	When encountering difficulties or adversity at work, every member of your team always prioritizes the interests of the team over personal interests.	Collective efficacy	CRTRQ CREST
6	When encountering difficulties or adversity at work, your team can bear the pressure.	Collective efficacy	CRTRQ
7	Your team can withstand the suffering of setbacks and adversity in work.	Collective efficacy	CRTRQ
8	When encountering difficulties or adversity at work, you can get help from other team members.	Mastery approaches	HMTRS
9	Your team can learn from adversity and crisis, thus achieving better development.	Mastery approaches	HMTRS
10	Every team member can adjust their approaches timely to overcome obstacles.	Mastery approaches	HMTRS
11	When encountering difficulties or adversity at work, your team will seek creative ways to change the situation.	Mastery approaches	HMTRS, ETRS
12	When encountering difficulties or adversity at work, every team member can understand and trust each other.	Social capital	HMTRS
13	Every member of your team can contribute diverse perspectives and experiences to work through collective wisdom.	Social capital	HMTRS
14	Every team member can accurately recognize team behavioral standards.	Group structure	HMTRS
15	Every team member is clear about acceptable and unacceptable ways of behavior.	Group structure	CREST
16	Every team member recognizes each other’s ways of behavior.	Group structure	HMTRS

Note: CRTRQ, Crisis Rescue Team Resilience Questionnaire (Liang, Shi, Liu & Gao, 2013); CREST, Characteristics of Resilience in Sports Teams Inventory (Decroos et al.,2017); HMTRS, Hierarchical and Multidimensional Team Resilience Scale (Sharma & Sharma,2016); ETRS, Entrepreneurial Team Resilience Scale (Blatt,2009)

#### Content validity

Content validity was used to identify whether the scale items adequately covered all aspects of the construct being measured [32]. Expert consultation is a suitable technique for evaluating the content validity of research instruments, which can convert individual viewpoints into consensus. Thus, we conducted double expert consultation to to assess the validity of the content of the initial NTRS. Nine experts were invited to take part in the consultation who had various clinical backgrounds and research fields that were closely related to team resilience, such as nursing psychology, nursing management, emergency nursing, and so on. Besides all experts were advanced professionals with graduate degrees. The experts’ detailed information is described in Table S1. The relevance, appropriateness, exhaustiveness, and disagreements of the items were consulted by mail. The experts were asked to evaluate whether each item is related to team resilience as well as to make ideas for components of NTRS with a 4 point Likert scale that ranged from “irrelevant” to “very relevant”. The scale-content validity index (S-CVI) and item-content validity index (I-CVI) were used to evaluated the degree of expert agreement [24]. In consideration of the number of experts, it is recommended in the literature that a minimum for the S-CVI and I-CVI were set at 0.90 and 0.78 [33].

### Phase 2: Scale Development

#### Pre-testing

To identify ambiguities and linguistic issues from the perspective of the target population, a two-round cognitive interview in Chinese with 11 nurses was conducted as a pre-test through video chat using Tencent Meeting which offered a reliable online conferencing service. We adopted the maximum difference method and selected nurses for interviews with different educational levels, work experience, workplaces, and professional titles. Demographic characteristics were shown in Table S1. Participants were first asked to fill out a questionnaire at Wen Juan Xing (online survey tool, https://www.wjx.cn), which was used as a both data collector and timer. And then we adopted the thinking-aloud method to discover whether the participants understood the meaning of the questionnaire [34]. At the same time, the interviewer documented the comments. Based on the results, we corrected items that were too difficult and too abstract for the interviewees to understand. Then the second round was conducted, and no further revisions were made based on its positive feedback.

### Phase 3: Scale Evaluation

#### Participants and sample size

A descriptive cross-sectional design was used in this stage. The study subject was comprised of nurses from a hospital in Shanghai collected as the study population by convenience sampling method. Participants who met the following criteria were included: (i) A nurse licensed to practice; (ii) working in this hospital for at least 1 year and have experienced or are experiencing a public health emergency event; (iii) agreeing to participate in this study.

The preview study has recommended sample size was 200–300 [23], whereas the required heterogeneous sample size required at least 300 participants as a basic requirement to support the analysis described.

### Ethical considerations

All the procedures of this study were in accordance with the Declaration of Helsinki and were approved by the Ethics Committee of the Shanghai Jiao Tong University School of Medicine (NO: SJUPN-202,020). The purpose and procedure of the study is explained to all participants. All of them participated in this study voluntarily and were promised to assure the anonymity of the data. Informed consent was obtained from all study participants after the participants received a written explanation of the study objectives.

### Data collection

From July 14, 2022, to August 15, 2022, we used a self-administered online questionnaire through Wen Juan Xing to collect data. The study population completed the questionnaire voluntarily and anonymously.

### Assessing validity

The construct validity of items was determined by exploratory factor analysis (EFA) and confirmatory factor analysis (CFA). An EFA was performed to examine factor structure. The Kaiser-Meyer-Olkin (KMO) measure of sample adequacy (≥ 0.80) and the Bartlett’s sphericity test (*P* < 0.05) indicated the adequacy of factor extraction [24, 35, 36]. In the EFA, the number of extracted factors was determined using a scree plot, eigenvalues greater than 1, and factor weights greater than 0.40 [25, 37]. We then performed a CFA to assess construct validity and confirm factor structure. The goodness of fit index, adjusted goodness of fit index, normed fit index, comparative fit Index values above 0.90, and the root mean squared error of approximation values from 0.08 or less indicates good model fitness [25, 38]. Furthermore, construct validity was performed to test whether the items consistently measure the factors indicating that the extracted mean variance (AVE) is greater than 0.70 and the composite reliability (CR) is greater than 0.50 that the construct validity is good [39, 40].

### Assessing reliability

In this study, the reliability of internal consistency was assessed using Cronbach’s alpha coefficients [35]. Cronbach’s alpha ranges from 0 to 1, with Cronbach’s alpha above 0.70 being considered “acceptable” and above 0.80 being “good” [23–25, 41].

### Statistical analysis

The general characteristics of the participants were examined using descriptive analysis. First, a measurement of the adequacy of the KMO sample selection and Bartlett’s test for sphericity were performed to ensure that the data collected were suitable for factor analysis. Subsequently, EFA and CFA were performed to assess factor structure and construct validity as mentioned above. Finally, Cronbach’s alpha was calculated to assess the internal consistency of the scale items. All analyzes were performed using the IBM Statistical Package for the Social Sciences version 24.0 (IBM Corp: Armonk, New York).

## Results

### Phase 1: Item Development

The content validity of the 16 items generated was assessed by nine experts in two rounds of correspondence. In the first round, 1 of 16 items (6.25%) scored below 0.78 on the I-CVI (0.67-1.00). The mean S-CVI score was 0.92. Based on the above results, 5 items were eliminated, 3 items combined, 2 items corrected and adjusted to 8 items as shown in Table 2. No I-CVI elements were found after the second round was less than 0.78, and the mean S-CVI score was 0.99, showing very good validity as shown in Table 2. After two rounds of consultation, a first draft of the 8-item questionnaire was developed, as shown in Table S2.

**Table 2:** Content validity results and suggestions from a two-round expert consultation

No.	I-CVI (First round)	Suggestions from experts	I-CVI (Second round)
1	0.89	This item was similar to item No.2, and experts suggested to combine them.	-
2	1.00		1.00
3	0.78	This item was advised to combine with item No.9.	-
4	0.78	This item was irrelevant to the scale.	-
5	0.67	This item was irrelevant to the scale.	-
6	1.00		1.00
7	1.00		1.00
8	1.00		1.00
9	1.00		1.00
10	1.00	This item was similar to item No.11, and experts suggested to delete it.	-
11	1.00		1.00
12	1.00	The words “every member in your team” was too absolutely.	1.00
13	0.89	This item was similar to item No.10 and No.11, and experts suggested to delete it.	-
14	1.00	This item was similar to item No.15 & 16, and experts suggested to combine them.	-
15	0.89	This item was similar to item No.14 & 16, and experts suggested to delete it.	-
16	0.78	Experts suggested to combine this item with item No.15 & 16 and revise it.	0.89

Note: I-CVI, Item-Level Content Validity Index; S-CVI: Scale-Level Content Validity Index

### Phase 2: Scale Development

As a pretest, two-stage cognitive interviews were conducted with 11 nurses to identify ambiguities and language problems from the perspective of the target group. The demographic characteristics are shown in Table S1. The average time to complete the questionnaire was 3.5 min. Participants experienced no discomfort with each question and no recommendations for stair design, length, and placement, nor did they feel uncomfortable with each question. After confirming the results of the cognitive interview, the language problems of 2 items were corrected and the tone of all items was adjusted. The detailed information was displayed in Table S2. Finally, the 8-item questionnaire was developed and there are five possible answers by a 5 point Likert scale (from disagree’=1 to ‘strongly agree’=5). Total scores range from 8–40 and each item scores are 1–5, with higher scores indicating greater nurse team resilience. A total scores greater than 24 indicates that the nurse team has high resilience.

### Phase 3: Scale Evaluation

#### Participant demographics

The sample included 421 nurses, of whom 409 (97.15%) were female, with a mean age of 32.57 years (SD 7.56) and more than half of the nurses were married. The average working experience was 11.10 years (SD 8.60 years). More than 75% of nurses had a bachelor’s degree or above. The detailed demographic characteristics were described in Table S3.

#### Validity

First, KMO (0.940) and Bartlett’s sphericity test (*χ*^2^ = 2818.254 and *p* < 0.0001) indicate that factor analysis is reasonable [40]. Then the EFA was performed and a common factorial solution was found. Figure S1 shows a screenshot of the characteristic values of the factors extracted in the EFA. The scree plot clearly showed curvature over a factor of 2. Only one factor had an eigenvalue greater than 1 (5.786). The extracted factor explained 72.330% of the common variance. Table 3 shows the factorial loadings of the eight NTRS elements for this single factor. The eight positions were loaded between 0.811 and 0.895 for the single factor extracted, as shown in Table 3. The CFA was then performed on the 8-item NTRS to test its fit to the data. The CFA presented the result as shown in Table 3, and the model shows a relatively good fit. All standardized factor loadings were greater than 0.40 [25, 37], and all paths were statistically significant (*p* < 0.05). The AVE of each factor was 0.679 and the CR was 0.944, indicating that the construct validity was determined as good.

**Table 3:** Description statistics and factor loading of 8 Items (n = 421)

Items	Mean	SD	Cronbach’s alpha if Item Deleted	EFA	CFA
1. Your team must be able to accomplish work goals efficiently.	4.50	0.58	0.936	0.863	0.854
2. When your team have some difficulties at work, it must be able to take on pressure.	4.45	0.64	0.937	0.848	0.835
3. Your team must be able to withstand frustration and adversity.	4.34	0.69	0.939	0.828	0.797
4. When your team have some difficulties at work, you must be able to get help from other team members.	4.53	0.61	0.939	0.813	0.749
5. Your team must be able to learn from adversity and crisis to become better.	4.44	0.67	0.940	0.811	0.742
6. When your team have some difficulties at work, every team member must be able to adjust their approaches timely to change or overcome it.	4.48	0.58	0.934	0.886	0.877
7. When your team have some difficulties at work, every team member must be able to understand and trust each other.	4.52	0.61	0.933	0.895	0.885
8. Every team member must be able to accurately understand the standards for their behavior and agree about how members are expected to behave.	4.48	0.61	0.936	0.856	0.840
Total % variance explained				72.330	
AVE					0.679
CR					0.944

Note: Model fitness: χ^2^ = 45.820, *p* = 0.001, χ2/df = 2.412, GFI = 0.974, AGFI = 0.950, NFI = 0.984, CFI = 0.990, RMSEA = 0.058. Note. AVE, average variance extracted; CR, composite reliability; GFI, goodness of fit index; AGFI, adjusted goodness of fit index; NFI, normal fit index; CFI, comparative fit index; RMSEA, root mean square error of approximation

#### Reliability

The internal consistency reliability was assessed using Cronbach’s alpha coefficients (Table 3). In this study, the Cronbach’s alpha was 0.944, with values greater than 0.80 being considered “good” [23, 42].

## Discussion

This study describes for the development and psychometric assessment of NTRS in the context of a public health emergency. The NTRS was based on that of Morgan and Sharma et al. proposed four-factor team resilience model and was developed using previous scales, the literature review, experts’ validations, and cognitive interviews. An eight items scale with one factor structure was developed and validity and reliability of the NTRS have been validated This sacle has shown a good validity and reliability.

It is shown that the results demonstrated NTRS was a reliable instrument to measure team resilience. There are many benefits and innovations of NTRS. Firstly, although many scales about team resilience have been validated, limited measures have been studied and applied to nursing staff. NTRS is the first specific instrument for nursing staff. According to the literature review, there are only one two scales that have been measured in nurse teams, the Crisis Rescue Team Resilience Questionnaire [14] and the Developing Adaptability and Performance in Teams to Enhance Resilience (ADAPTER) Scale [9]. The former measure has been developed in the context of emergencies for crisis rescue staff and applied it to nurse teams in the context of Covid-19 [43]. The ADAPTER scale is designed for company employees by van der Beek et al. from Netherlands [9] and has been translated into Chinese version to measure team resilience among nurses [13]. Secondly, most of the existing team resilience scale is so complex that has poor practicality. The Hierarchical and Multidimensional Team Resilience Scale (HMTRS) has four primary dimensions and 10 sub-dimensions with a total of 50 items. Although HMTRS has been applied by cancer healthcare professionals in France to evaluate their team resilience, it is too complex and time-consuming to complete in terms of nursing [10]. Besides Resilience at Work Team Scale is the same way [12]. Therefore, in consideration of the time-consuming and costly nature of psychometric research, the length of NTRS wasn’t as same as other scales about team resilience, we have combined similar content and compressed the length of the scale. The NTRS scale comprises eight items is a useful self-report questionnaire for measuring resilience of the nurse team. The score with higher scores indicating greater nurse team resilience. A total scores greater than 24 indicates that the nurse team has high resilience, and a total scores less than or equal to 24 indicates that it is low and needs to be improved. Team leaders can analyze the problems existing in the team’s resilience based on the specific scores of each item, and accurately improve the team’s resilience. Additionally, a team’s resilience is dynamic and can change over time as adversity is overcome, NTRS may help researchers find out the occasion when the nursing team resilience is at the lowest level. But our study hasn’t verified its validation in the longitudinal study, indicating that further study can start from this aspect.

### Limitation and strengths

There are some limitations in NTRS development and validation. Firstly, in the scale evaluation process was limited the sample to a single hospital despite the high response rate and the large sample size. Secondly, this scale has not been verified for calibration validity because there is a lack of a gold standard for the evaluation of the resilience of the nurse team. Finally, besides this scale has not been verified its validation in the longitudinal study, indicating that further study can start from this aspect. Further testing of the scale on a wider variety of healthcare professionals in more study sites is important to gain more confidence in the scale’s reliability and validity for international use. Additional, to facilitate the clinical use of this scale, future research should also consider the visual development of the scale.

Although the epidemic has shown the important role of nurses in the healthcare system, we should consider the collective trauma that caregivers have experienced during the pandemic [44]. Thus, nurse team resilience, issues that need to be addressed particularly within global nursing workforce crisis. Developing a good team resilience scale will enable future more resilient in health systems as specific to nurse team in context of public health emergencies setting. The NTRS can assess team resilience in nurses that nursing education and management resources can be allocated to improve policies and training programmes to effectively support nurses. Moreover, this instrument able to help nursing teams respond positively to adversity at work, thereby serving as a starting point for improving team resilience and helping organizations develop strategies that may improve team efficiency and stability.

## Conclusions

This study developed a scale with good reliability and validity to measure the resilience of nursing teams. This scale can be used as an assessment tool to identify team resilience and help nursing teams respond positively to adversity at work and improving team resilience, thereby helping organizations develop strategies that may improve team efficiency and stability.

### Electronic supplementary material

Below is the link to the electronic supplementary material.

**Supplementary Material 1**: Table S1. Demographics information about experts and cognitive interview participants. Table S2. Final Development of the Instrument. Table S3. Distribution of demographic characteristics. Figure S1. Scree plot obtained from EFA for the scale.

## Data Availability

The datasets generated and/or analysed during the current study are not publicly available due to its ethical concerns, supporting data cannot be made openly available, but are available from the corresponding author on reasonable request.
