# Supplementary material for: Development and validation of the Nurse Team Resilience Scale (NTRS) in the context of public health emergencies
Source: BMC Nurs. 2023 Dec 20;22:489. doi: 10.1186/s12912-023-01627-9 (PMC10731786; doi:10.1186/s12912-023-01627-9)
Supplement: Supplementary file 1 — Supplementary Material 1: Table S1. Demographics information about experts and cognitive interview participants. Table S2. Final Development of the Instrument. Table S3. Distribution of demographic characteristics. Figure S1. Scree plot obtained from EFA for the scale. [file 12912_2023_1627_MOESM1_ESM.docx]

**Table S1.** **Demographics information about experts and cognitive interview participants**

| **Demographics information about experts (n = 9)** | | | | |
| --- | --- | --- | --- | --- |
| **No.** | **Educational level** | **Work experience** | **Research field** | **Professional title** |
| **1** | Master's degree | 10 | Nursing psychology | Deputy chief nurse |
| **2** | Master's degree | 13 | Nursing management | Deputy chief nurse |
| **3** | Master's degree | 17 | Nursing education | Deputy chief nurse |
| **4** | Master's degree | 19 | Nursing management,  Nursing education | Deputy chief nurse |
| **5** | Master's degree | 20 | Emergency nursing,  Nursing management | Chief nurse |
| **6** | Master's degree | 26 | Nursing education | Deputy chief nurse |
| **7** | Doctor’s degree | 10 | Emergency nursing | Associate Professor |
| **8** | Doctor’s degree | 19 | Infection control，  Nursing management | Chief nurse |
| **9** | Doctor’s degree | 26 | Mental health | Deputy chief nurse |
| **Demographics information about cognitive interview participants (n = 11)** | | | | |
| **No.** | **Educational level** | **Work experience** | **Workplace** | **Professional title** |
| 1 | Junior college | 3 | Designated isolation hospital | Nurse |
| 2 | Junior college | 3.5 | Designated isolation hospital | Nurse |
| 3 | Junior college | 5 | Designated isolation hospital | Nurse |
| 4 | Bachelor's degree | 1 | Square cabin hospital | Nurse |
| 5 | Bachelor's degree | 1.5 | Square cabin hospital | Senior nurse |
| 6 | Bachelor's degree | 5 | Hospital emergency room | Senior nurse |
| 7 | Bachelor's degree | 8 | Designated isolation hospital | Senior nurse |
| 8 | Bachelor's degree | 11 | Nucleic acid sampling team | Charge nurse |
| 9 | Master's degree | 8 | Public health center | Charge nurse |
| 10 | Master's degree | 8 | Designated isolation hospital | Charge nurse |
| 11 | Master's degree | 10 | Designated isolation hospital | Charge nurse |

**Table S2. Final Development of the Instrument**

| **No.** | **First draft** | **Results about cognitive interview** | **Final Items** |
| --- | --- | --- | --- |
| 1 | Your team can accomplish work goals efficiently. | “can” was changed to “must be able to” | Your team must be able to accomplish work goals efficiently. |
| 2 | When your team have some difficulties at work, it can take on pressure. | “can” was changed to “must be able to” | When your team have some difficulties at work, it must be able to take on pressure. |
| 3 | Your team can withstand frustration and adversity. | “can” was changed to “must be able to” | Your team must be able to withstand frustration and adversity. |
| 4 | When your team have some difficulties at work, you can get help from other team members. | “can” was changed to “must be able to” | When your team have some difficulties at work, you must be able to get help from other team members. |
| 5 | Your team can learn from adversity and crisis so as to become better. | “can” was changed to “must be able to” | Your team must be able to learn from adversity and crisis so as to become better. |
| 6 | When your team have some difficulties at work, it can look for creative solutions to alter difficult situations. | “can” was changed to “must be able to”; the interviewers didn’t understand the "creative solutions" and couldn’t give examples, but they believed that "creative solutions" did exist in the work. It is recommended to modify it to “adjust their approaches”. | When your team have some difficulties at work, every team member must be able to adjust their approaches timely to change or overcome it. |
| 7 | When your team have some difficulties at work, every team member can understand and trust each other. | “can” was changed to “must be able to” | When your team have some difficulties at work, every team member must be able to understand and trust each other. |
| 8 | Every team member agrees about how members are expected to behave. | “can” was changed to “must be able to”; The interviewers have different understanding of the "behavioral standards", and even the same as the skill operation specifications. It is recommended to change to “the standards for their behavior”。 | Every team member must be able to accurately understand the standards for their behavior and agree about how members are expected to behave. |

**Table S3. Distribution of demographic characteristics (n = 421)**

|  | **n（%）** |
| --- | --- |
| **Age(years)** |  |
| Mean ± SD | 32.6 ±7.6 |
| **Work experience（years）** |  |
| Mean ± SD | 11.1±8.6 |
| **Sex** |  |
| Women | 409 (97.1) |
| Men | 12 (2.9) |
| **Marital status** |  |
| Married | 243 (57.7) |
| Unmarried | 173 (41.1) |
| Divorced or Widow | 5 (2.9) |
| **Educational level** |  |
| Junior college | 102（24.2） |
| Bachelor's degree | 306 (72.7) |
| Master's degree or above | 13 (3.1) |
| **Professional title** |  |
| Nurse | 73 (17.3) |
| Senior nurse | 273 (64.8) |
| Charge nurse | 65(15.4) |
| Deputy chief nurse or above | 10(2.3) |
| **Team Responsibility** |  |
| Head of the team | 18 (4.3) |
| Group leader | 17(4.0) |
| Team member | 386(91.7) |


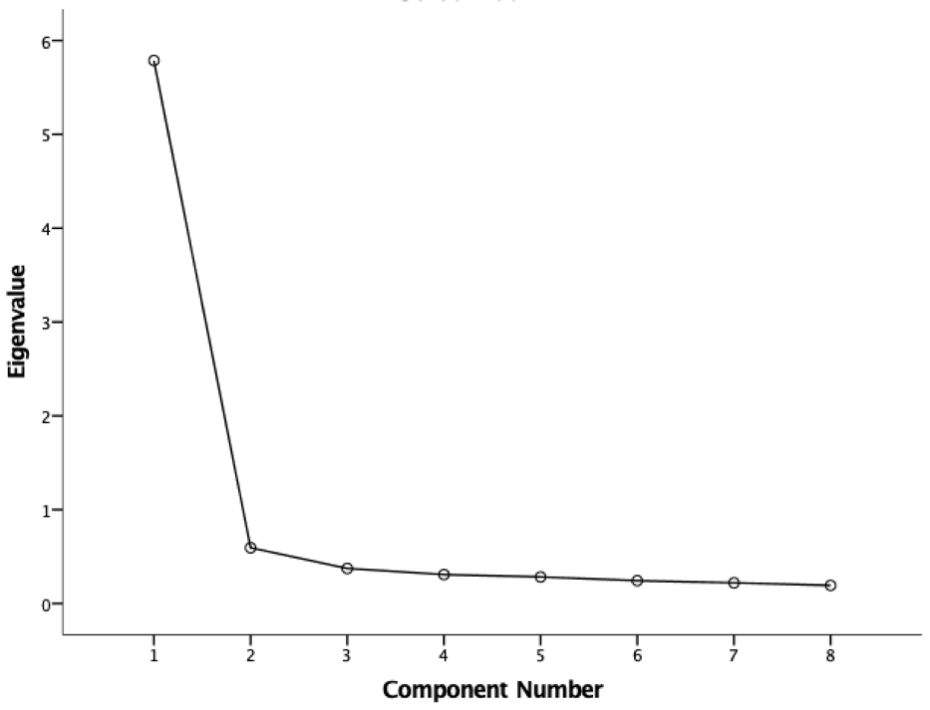


**Figure S1. Scree plot obtained from EFA for the scale**
